# Dislocation driven nanosample plasticity: new insights from quantitative *in-situ* TEM tensile testing

**DOI:** 10.1038/s41598-018-30639-8

**Published:** 2018-08-13

**Authors:** Vahid Samaee, Riccardo Gatti, Benoit Devincre, Thomas Pardoen, Dominique Schryvers, Hosni Idrissi

**Affiliations:** 1 Electron Microscop for Materials Science (EMAT), Department of Physics, University of Antwerp, Antwerp, Belgium; 2 Laboratoire d’Etude des Microstructures, UMR104 CNRS-ONERA, 29 av. de la division Leclerc, Chatillon, France; 3 Institute of Mechanics, Materials and Civil Engineering, Université catholique de Louvain, Louvain-la-Neuve, Belgium

## Abstract

Intrinsic dislocation mechanisms in the vicinity of free surfaces of an almost FIB damage-free single crystal Ni sample have been quantitatively investigated owing to a novel sample preparation method combining twin-jet electro-polishing, *in-situ* TEM heating and FIB. The results reveal that the small-scale plasticity is mainly controlled by the conversion of few tangled dislocations, still present after heating, into stable single arm sources (SASs) as well as by the successive operation of these sources. Strain hardening resulting from the operation of an individual SAS is reported and attributed to the decrease of the length of the source. Moreover, the impact of the shortening of the dislocation source on the intermittent plastic flow, characteristic of SASs, is discussed. These findings provide essential information for the understanding of the regime of ‘dislocation source’ controlled plasticity and the related mechanical size effect.

## Introduction

Submicron crystals with sizes ranging from several hundred nanometers to a few micrometers are widely used for micro-electro-mechanical systems (MEMSs) and other small-scale devices^1^. The unique properties of such small-scale materials are also very promising for extremely strong and ultra-light nano-lattices and metamaterials^2^. Thin films used in a variety of technologies are polycrystals made of submicron crystals with only one grain in the thickness direction. This motivated several research groups to investigate the mechanical properties and elementary deformation/failure mechanisms in these systems^3–5^. Extensive experimental research has shown that the yield strength of submicron materials depends inversely on size^3,6–20^. Although “smaller is stronger” leads to beneficial performance of submicron single crystals, it has been shown that plastic deformation at that length scale is usually intermittent and strongly heterogeneous^21^ which can cause problems in the fabrication and design. Considering the fact that, in small-scale materials, the sample size is comparable to the characteristic length of the dislocation microstructure, understanding the discrete properties of dislocations becomes of paramount importance.

Since the advent of transmission electron microscopy (TEM), intensive efforts have been made to reveal the fundamental plasticity mechanisms in different classes of inorganic materials. Development of *in-situ* TEM straining holders allowed real time investigation of microstructure evolution under various mechanical loading modes. Legros *et al*.^12^ recently reviewed different types of *in-situ* TEM mechanical testing techniques and listed the advantages and disadvantages. The main disadvantage of classical *in-situ* TEM holders, which was the lack of quantitative data, has been recently solved through designing miniaturized micro-electro-mechanical-systems (MEMS) adapted to TEM holders. The new MEMS-based holders enable various accurate quantitative mechanical testing configurations, including indentation, compression, bending and tension on well-designed small-sized sample shapes^8,10,16,18,22–33^. This has opened new paths to investigate the origin of mechanical size effects in small scale samples in a quantitative manner as well as to reveal the origin of the intermittent plastic regime.

In order to reveal the underlying dislocation mechanisms causing size effects observed in submicron crystals, several physical scenarios have been proposed. Among them, the dislocation starvation (i.e., dislocation annihilation at free surfaces) and the dislocation source truncation (i.e., single arm source) models are by far the most widely accepted^3,7–20^. However, quantitative experimental characterization of the direct correlation between the single arm source (SAS) mechanism and the submicron plastic behaviour is still very limited. Furthermore, a general understanding of the evolution of the flow stress after the onset of yielding is still lacking. For example, several experimental studies reported contradictory data regarding the presence of strain hardening in submicron FCC single crystals under uniaxial compression^9,34–37^. Some researchers also highlighted the role of external conditions such as focused ion beam (FIB)-damage on the mechanical size effect. Indeed, the micron-sized samples used for *in-situ* TEM mechanical testing are often prepared by FIB for its fast, reliable, and accurate micron-level machining ability^25,38^. Nevertheless, FIB-induced defects at sample surfaces not only can hamper proper imaging of the microstructure, but also they can change the deformation mechanisms and the mechanical responses of the pristine material^38–42^.

To the best of authors’ knowledge, there is only a relatively small body of literature in which few other methods such as selective etching have been used to avoid FIB for sample preparation^16,19,29,30,43–45^. However, because these methods are not site-specific, they cannot be used to generate samples with preselected microstructure and/or crystallographic orientation. Some researchers have also used heat treatment to remove FIB induced damages before micro/nanomechanical testing^8,27,46–48^. However, heat treatment of FIB samples or selective etching were mainly used for *in-situ* TEM/SEM compression of pillars or *in-situ* SEM tensile experiments while quantitative *in-situ* TEM tensile testing on FIB defect-free single-crystal samples is still missing in the literature. Also, micro tension is generally used to overcome most of the experimental shortcomings of the micropillar approach such as the deformation of the substrate and the lateral constraint between pillar top surface and compression tip that can affect the nucleation and evolution of dislocations as well as the recorded stress-strain curves^49^.

Recently, Samaeeaghmiyoni *et al*., combined twin-jet electro-polishing and FIB in order to minimize the amount of FIB damages in Ni specimens dedicated to quantitative *in-situ* TEM tensile testing^22^. Twin-jet electro-polishing was used for thinning the samples to allow electron transparency and FIB was used just for cutting the sample edges and for mounting the sample on the TEM holder. However, in spite of an overall decrease of FIB damages in the middle of the samples, the edges were still highly damaged by the FIB cut as evidenced by implantation of Ga ions as well as by the presence of a high density of FIB-induced dislocation loops with different sizes. These loops were observed to act as sources for the nucleation of mobile dislocations upon *in-situ* TEM straining. Strain hardening due to the opening of dislocation loops with decreasing size was also observed. The objective of the present work is to contribute to a better understanding of the plasticity mechanisms by using Ni samples not affected by preparation induced extrinsic artefacts. Thus, we used the aforementioned preparation method and added an *in-situ* TEM annealing step prior to *in-situ* tensile testing to anneal out the remaining FIB induced defects. Comprehensive quantitative observation of the intrinsic discrete dislocation mechanisms under dedicated imaging conditions was performed to investigate the origin of the size dependent intermittent plasticity of single crystals.

**Figure 1:**
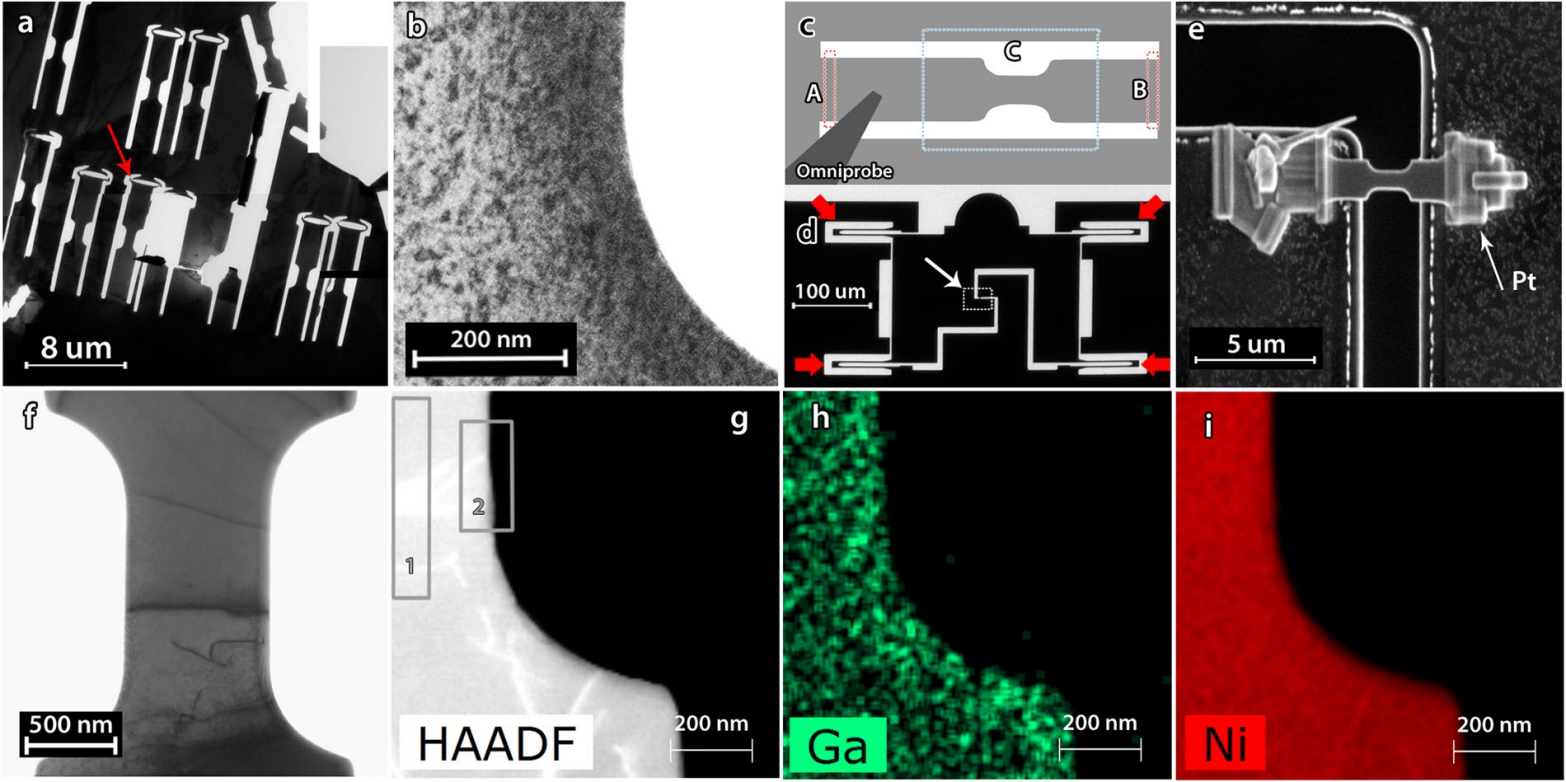
TEM BF image of (**a**) 3-mm electro-polished sample after FIB cutting, (**b**) the edge of a dog-bone showing FIB induced defects. (**c**) A schematic of a dog-bone sample showing the location where the Omniprobe attaches to the sample, the areas A and B which will be cut after the annealing and the area C which should be kept unexposed during mounting. (**d**) TEM image of a PTP device, (**e**) SEM image showing a final configuration of a dog-bone on a PTP device, (**f**) TEM-BF image showing a near dislocation-free sample after annealing and mounting on the PTP device, (**g**) ADF-STEM image), (**h**) and (**i**) EDX chemical maps of Ni, and Ga, respectively, displayed as counts.

Dog-bone shaped samples dedicated for quantitative *in-situ* TEM tensile testing were prepared from 3 mm electro-polished pure Ni discs, see Fig. 1a. In Fig. 1b, a TEM bright-field (BF) image of the dog-bone sample edge before heat treatment, shows FIB induced defects. The specimen was first annealed *in-situ* in the TEM, then mounted on a dedicated silicon Push-to-Pull (PTP) device in order to perform quantified *in-situ* TEM tensile testing with the commercial PI95 PicoIndenter instrument (Brucker.Inc)^50^, see Fig. 1c,e. Bight-field TEM as well as energy dispersive X-ray Spectroscopy (EDX) revealed that the extra *in-situ* TEM annealing step (5 min at 400 °C, 10 min 500 °C and 60 min at 700 °C) prior to *in-situ* tensile testing removed the FIB damages. This can be seen in Fig. 1f,g,h,i showing BF-TEM, ADF-STEM and STEM-EDX maps for Ga and Ni, respectively. The measured Ga amount in the selected grey rectangles, 1 and 2, as well as the whole area in Fig. 1g are 0.41 at.%, 0.78 at.%, and 0.73 at.%, respectively, which are less than the EDAX method accuracy of 1 at.%. Further details about the sample preparation and the *in-situ* TEM straining can be found in the Methods section.

## Results and Discussion

**Figure 2:**
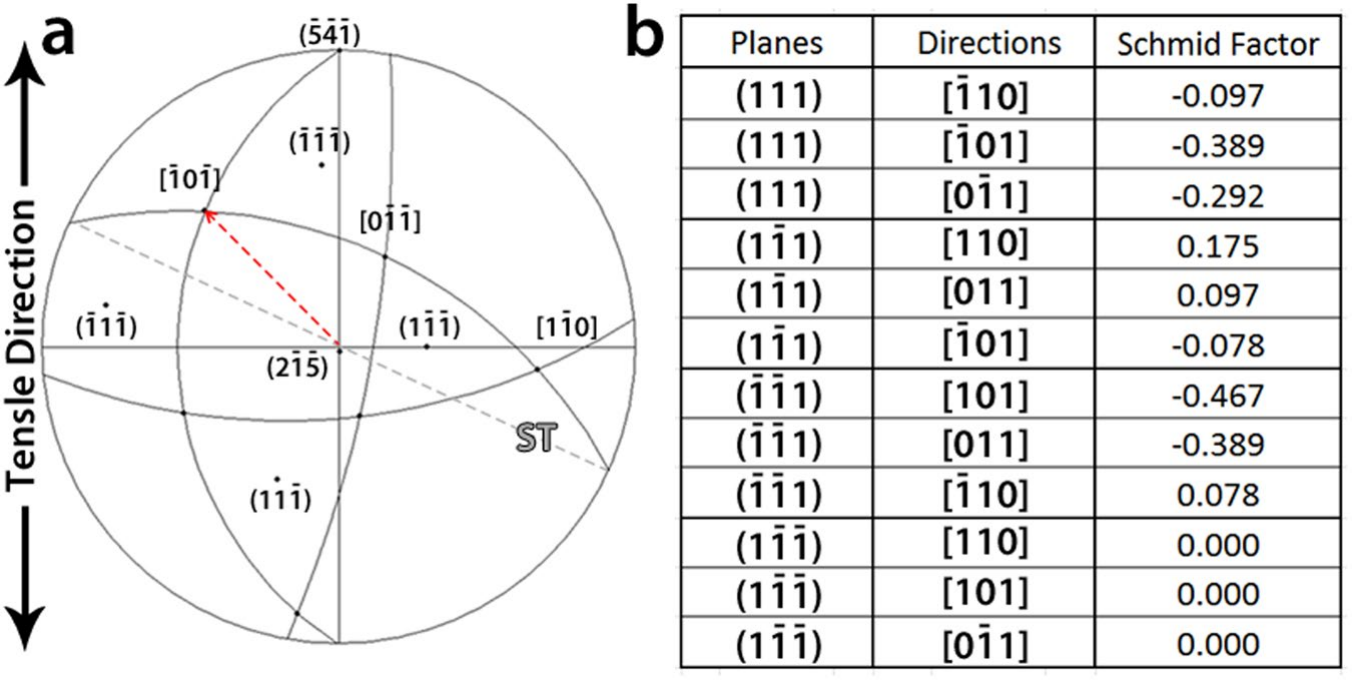
(**a**) Stereographic projection of the tensile sample and (**b**) Schmid factor values of slip systems in the sample.

Figure 2a exhibits the stereographic projection of the Ni crystal sample shown in Fig. 1f. The tensile direction and the normal to the surface were close to $[\bar{5}\,\bar{4}\,\bar{1}]$ and $[2\,\bar{1}\,\bar{5}]$, respectively. Schmid factors on the different slip systems are listed in Fig. 2b. The sample was oriented close to multiple slip conditions (the ratio of the Schmid factor of the secondary to the primary slip systems $\frac{{{\rm{\tau }}}_{s}}{{{\rm{\tau }}}_{p}}$ is 0.83). The red (dashed) arrow in Fig. 2a shows the Burgers vector with the highest Schmid factor while the gray dashed line indicates the trace of the $(1\,1\,\bar{1})$ plane. In Fig. 1f it can be seen that the initial microstructure consists of few tangled dislocations that survived the heat treatment. In the literature, both experiments and simulations have shown that the final dislocation structure after thermal relaxation of FIB defects is a 3D tangled network of dislocations containing mobile/immobile pinning points^14,15,27^.

**Figure 3:**
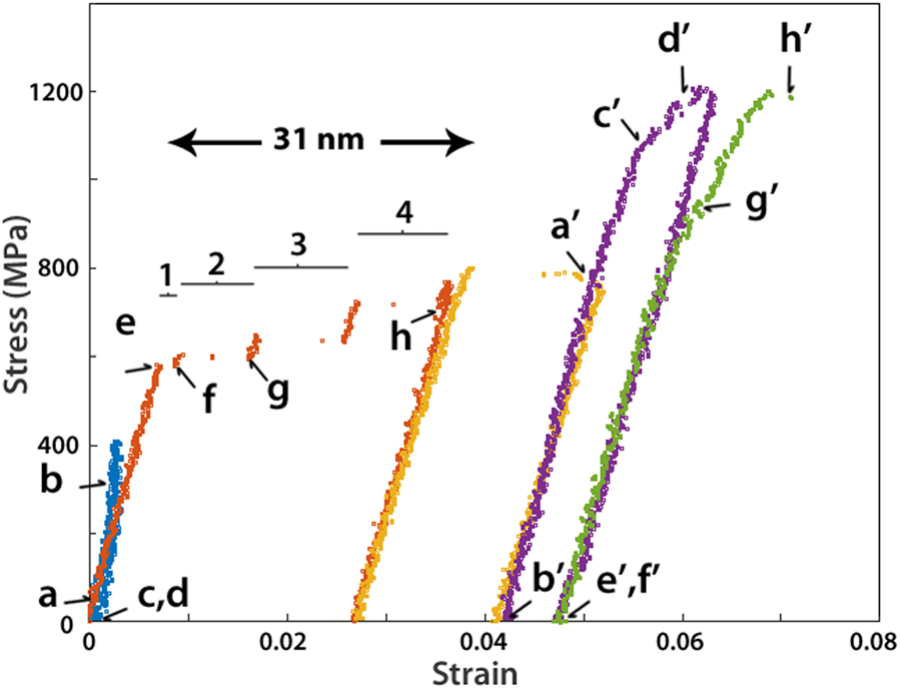
Engineering tensile stress-strain curves involving 5 loading cycles. Letters correspond to the snapshots shown in Figs 4 and 6. The numbers and segments indicate the strain bursts in cycle 2.

**Figure 4:**
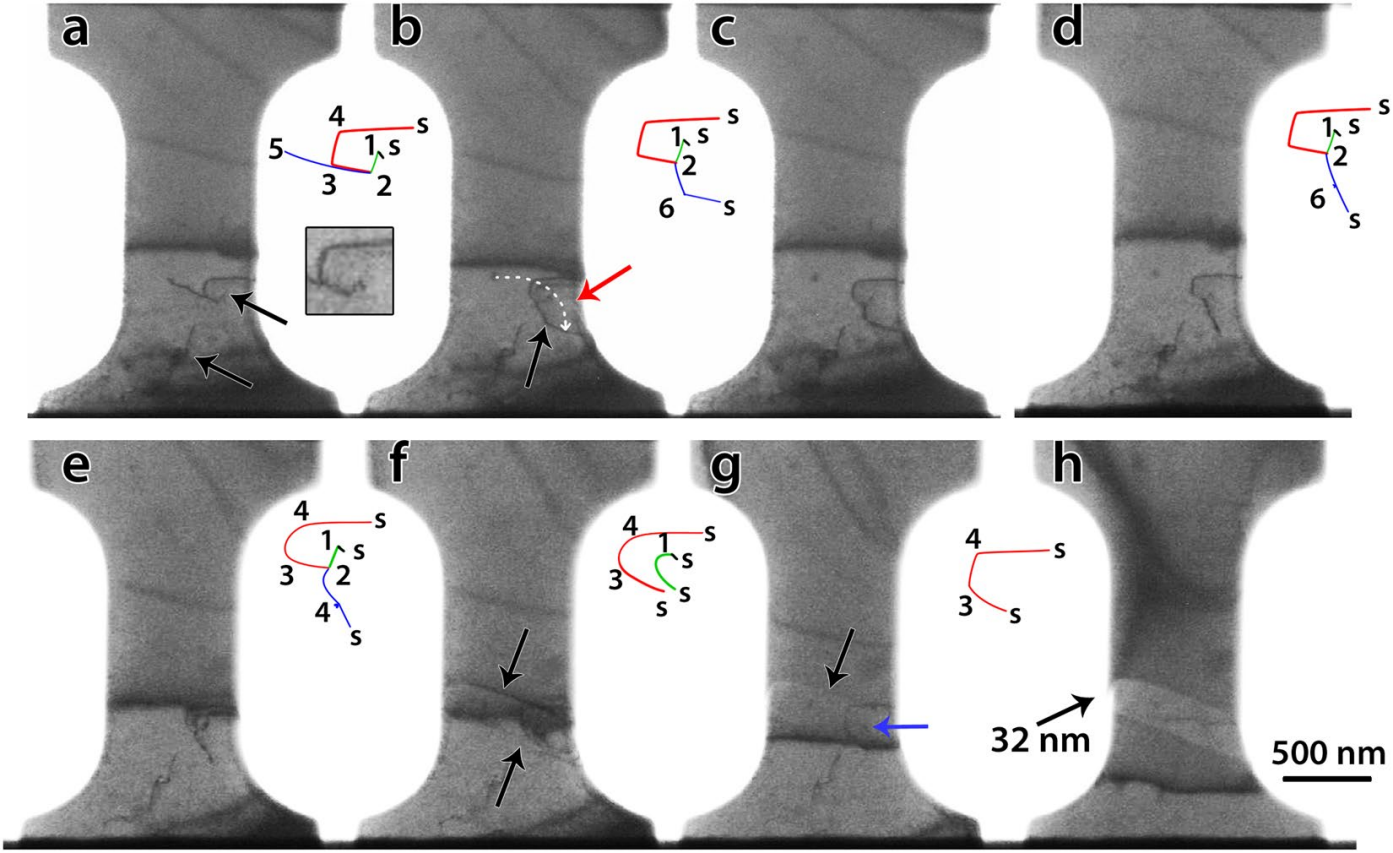
Snapshots from the *in-situ* TEM deformation movie during cycle 1 (**a**–**c**) and cycle 2 (**d**–**h**). Schematic configurations of dislocations were drawn to the right of some snapshots. The numbers indicate the position of pinning points due to debris or cross-slip of dislocations. The sites in which the dislocations emerge to the free surface are indicated by “s”. A magnified image of the initial dislocation configuration is shown in the right inset of (**a**).

The sample was subjected to 5 loading-unloading cycles with maximum stress equal to 420 MPa, 770 MPa, 800 MPa, and 1209 MPa while failure occurred during the last cycle at 1200 MPa. Figure 3 shows the stress-strain curves of the sequential tensile experiments. Note that, after each cycle, the experiment was stopped in order to set the parameters of the following cycle. During cycle 1 (blue plot in Fig. 3) the behaviour was elastic and relaxation of the initial dislocation structure was observed. Indeed, as depicted in Fig. 4b, the blue dislocation was unlocked from the debris at point 5 and glided around the pinning point 2 as indicated by the dashed arrow in Fig. 4b, before being pinned again at point 6 by another obstacle (black arrow in Fig. 4b) and the surface. The resulting configuration, including the left-over debris as a small black dot at point 5, can still be observed in Fig. 4c after unloading. These debris points are probably residual small sessile dislocation loops associated to point defect clusters generated by FIB^27,42^. The slip trace (ST) left by the glide of the blue dislocation (marked by the red arrow in Fig. 4b) indicates that this dislocation slips along the $(1\,1\,\bar{1})$ plane. During the setting of the parameters for cycle 2 (orange plot in Fig. 3b), the segment of the blue dislocation pinned between point 6 and the surface was lightly modified. It moved in its slip plane to decrease the dislocation length and therefore relaxed the source configuration (Fig. 4d).

In cycle 2, the sample started yielding at 580 MPa which is one order of magnitude higher than the typical yield stress reported in macroscopic Ni single crystal (~50 MPa^51^). The stress-strain response in Fig. 3 clearly exhibits discrete strain bursts separated by intervals of nearly elastic loading. The lack of points between the bursts is due to the sudden increase of displacement that cannot be captured in the movie used for accurately measuring the displacements. The loss of contact between the indenter and the PTP device can be excluded as responsible for such behaviour because no load drop is detected. In the following paragraphs, the origin and characteristics of the staircase behaviour in cycle 2 are discussed based on the TEM snapshots shown in Fig. 4.

In the BF image of Fig. 4e obtained just before yielding, bowing out of the dislocations can be observed. After yielding, the first strain burst occurred which was accompanied by the destruction of the dislocation configuration shown in Fig. 4e and the formation of STs parallel to the $(1\,1\,\bar{1})$ plane as indicated by black arrows in Fig. 4f. The new dislocation configuration consists of the red, green and black dislocations shown in Fig. 4f while the blue dislocation has disappeared. It is worth noting that the ST induced by the glide of the blue dislocation in cycle 1 (red arrow in Fig. 4b) is different from the ST shown in Fig. 4f (see SM). Furthermore, Fig. 4g confirms that the red dislocation remains immobile in cycle 2. This is probably due to the complex structure of this dislocation composed of segments lying in different planes as evidenced by the sharp angles between these segments in Fig. 4d. Thus, the most plausible scenario is that, after yielding, the pinning point 2 was destroyed by surface annihilation or unzipping^52^, leading to the formation of a single arm source 1 (SAS1) at the pinning point 1 (the green dislocation in Fig. 4f). Avalanches of green dislocations presumably nucleated from SAS1 to create the ST shown in Fig. 4f and the first strain burst in cycle 2. A magnified image of the SAS1 indicated by the blue arrow in Fig. 4g is shown in Fig. S4 in SM.

In Fig. 4g,h, the height of the ledge created by the glide of the green dislocations in the $(1\,1\,\bar{1})$ plane has increased after the successive strain bursts. This, as well as the nearly identical amount of plastic deformation induced after the strain bursts between ‘f’ and ‘h’ in Fig. 3, confirm that the bursts in cycle 2 were induced by the same SAS1. However, it should be noted that the first strain burst between points ‘e’ and ‘f’ in the same figure is smaller. This can be explained by the difficulty of the first dislocations to escape from the sample due to the presence of a very thin oxide layer at the surface. Indeed, the black diffraction contrast at the ST in Fig. 4f might indicate the presence of one or very few dislocations near the surface. The presence of dislocations pile-up at this position is unlikely since it cannot explain the occurrence of the first strain burst^53^. The black diffraction contrast at the ST in Fig. 4f cannot be observed in Fig. 4g,h, indicating that, with increasing deformation, the oxide layer ceased acting as an obstacle retarding the escape of dislocations at the free surfaces, a mechanism that has been reported before in thin films with native oxide layers^54^. Furthermore, because dislocation pile-ups were not observed before the macroscopic yielding in cycle 2 of Fig. 3, it can be concluded that such behaviour is mainly controlled by the nature/length of the initial dislocations involving possible segregation of Ga atoms on these dislocations. The role of a thin oxide layer at the surface cannot be considered as a dominant mechanism controlling the yield stress. This is also in agreement with the absence of clear observation of oxide layer at the surface using conventional high resolution TEM and electron energy loss spectroscopy (EELS) which can be attributed to the very small thickness of this layer.

Based on the total plastic displacement (31 nm in Fig. 3) and the height of the ledge (32 nm in Fig. 4h) induced by the SAS1 in cycle 2, it can be concluded that around 200 dislocations have been nucleated from this source in the active slip system $a/2\,[\bar{1}\,0\overline{\,1}]\,(1\,1\overline{\,1})$ (see SM for more details). Therefore, the observed stair case flow in cycle 2 of Fig. 3 is due to the intermittent operation of the SAS1 in the $a/2\,[\bar{1}\,0\overline{\,1}]\,(1\,1\overline{\,1})$ slip system. The stair case flow is often attributed to the sequential activation and deactivation of different SASs^5,14,15,24,55,56^. This interpretation, which makes sense in samples with high dislocation density (including FIB induced dislocations), is not consistent with the present observations. Thus, the present result offers the possibility to directly investigate plastic flow for individual SASs in quantitative manner.

**Figure 5:**
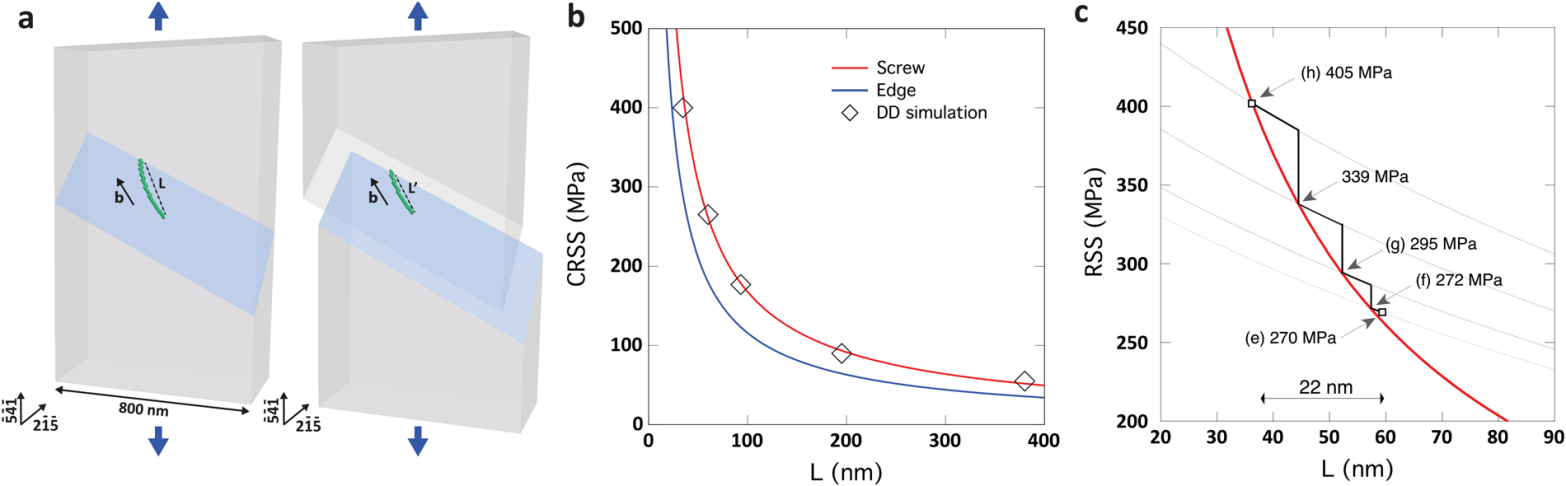
(**a**) Illustration from DD simulations showing the effect of the slipped area reduction on the SAS critical length after a strain burst. (**b**) CRSS vs. SAS length calculated with equation (1) for screw or edge dislocation character compared with DD simulation results when using the exact crystal geometry. (**c**) Schematic illustration of the resolved shear stress (RSS) variation in the slip plane of the SAS. The red curve is the equation (1) prediction for a screw dislocation and the black dashed lines are the RSS evolution (*τ*_*area*_) deduced when considering the slip area reduction effect at the end of the bursts in cycle 2 (see SM for more details).

Strain hardening due to source size reduction has been reported before by Chisholm *et al*.^11^. However, the elementary mechanisms controlling the formation and the operation of the individual SASs as well as the relationship between these mechanisms and the overall plastic flow have not been investigated. A deeper analysis of the TEM images in Fig. 4 helps us to understand the origin of the strain hardening accompanying the operation of the SAS1 in cycle 2 of Fig. 3. Indeed, it can be seen in this cycle that the stress increases after each strain burst with pure elastic deformation. Usually, in FIB-prepared samples, once dislocations emitted from the weakest SAS get blocked by the abundant FIB damages, the source is shut down by the exerted back stress or by the changes of the local stress state, and a second source starts to operate at higher stresses, leading to strain hardening^18,24^. In cycle 2 of Fig. 3, such scenario can be excluded as only one SAS is active. Furthermore, interactions of dislocations nucleated from SAS1 with other dislocations were not observed. In the following, the strain hardening observed in cycle 2 of Fig. 3 is explained by the decrease of the SAS1 length (the green segment in Fig. 4f) due to the decrease of the mobile dislocation glide area. Such effect is schematically illustrated in Fig. 5a. Efforts are thus made to quantify the decrease of the SAS1 length and to clarify its relationship with the intermittent plastic flow of this source.

Changes of the length of the SAS1 in cycle 2 can be determined by analysing the evolution of the critical stress needed to activate the source with the accumulated plastic displacement. The critical resolved shear stress (CRSS) to activate a SAS can be calculated using equation (1), modified from the known equation for a Frank-Read source by replacing the length *L* by *L*/2^57,58^.

1 $$CRSS=\frac{\alpha G{\boldsymbol{b}}}{4\pi l}\,(\mathrm{ln}\,(\frac{2l}{b})+1)\,$$

where *α* is a coefficient equal to 1 or 1/(1 − *v*) for edge or screw dislocations, respectively, *G* is the shear modulus (76 GPa), ***b*** is the magnitude of the Burgers vector (0.249 nm), Poisson ratio *v* is equal to 0.31 and *L* is the SAS length.

To support the predictions of equation (1), Dislocation Dynamics (DD) simulations^59^ were performed with the Discrete Continuous Model^60^, in order to match experimental loading conditions and to take into account SAS-surface interaction. As illustrated in Fig. 5a, DD simulations reproduced precisely the plastic deformation of the TEM sample. Different scenarios have been tested by changing the SAS1 location and length. Those computations established that the CRSS predicted with equation (1) is in very good agreements with DD simulations accounting for dislocation-surface elastic interactions (see Fig. 5b). Furthermore, in agreement with elastic theory predictions we confirmed that the SAS1 critical configuration is close to the screw dislocation orientation (Fig. 5b). Figure 5c exhibits the evolution of the resolved shear stress in the SAS1 slip plane as function of the source length during cycle 2. In this figure, the red curve is the equation (1) prediction for a screw dislocation. Except for the arrow (e) at 270 MPa that indicates the onset of yielding in cycle 2, the other arrows in Fig. 5c report the resolved shear stress at the end of each burst and which can be directly calculated using the Schmid factor from the engineering stress-strain curve reported in Fig. 3. These values have also been corrected by considering the slip area reduction effect at the end of the bursts in cycle 2 (see SM). Figure 5c also shows that the length of the source is reduced by ~22 nm in cycle 2. This value is in a very good agreement with the decrease of the source length of ~24 nm obtained by simply considering the slip geometry (see SM for more details).

These results show that two main competing mechanisms are controlling the dynamics of the SAS1 in cycle 2: (i) the reduction of the slipped area (i.e., softening due to the increase of the local applied stress *τ*_*area*_) and (ii) the decrease of the source length (i.e., strengthening due to the increase of the CRSS to activate the dislocation source). The scenario illustrated in Fig. 5c can thus explain the origin of the intermittent plastic flow of this source, based on the assumption that an immobile SAS needs a stress overshoot to be activated. In this figure, the black dashed lines indicate the evolution of *τ*_*area*_ deduced when considering the slip area reduction effect at the end of the bursts in cycle 2 (see SM). At the beginning of each strain burst, *τ*_*area*_ > *CRSS* and the SAS activation is made possible. Progressively, the increment of *τ*_*area*_ (Δ*τ*_*area*_) is not sufficient to allow further operation of the source and the SAS stops when (*τ*_*area*_ + Δ*τ*_*area*_) < (*CRSS* + Δ*CRSS*). Such competition between a softening and a strengthening mechanism explains the existence of intermittent flow. Here, one must notice that it is not possible to predict the amplitude of plastic bursts. Indeed, this calculation would require the measurement of the stress overshoot needed to activate the SAS. Such quantity is difficult to define since it is impacted by many features such as the loading control, anelastic pinning effects that might exist on immobile dislocations ending at a surface and internal stress sources neglected in our DD simulations.

**Figure 6:**
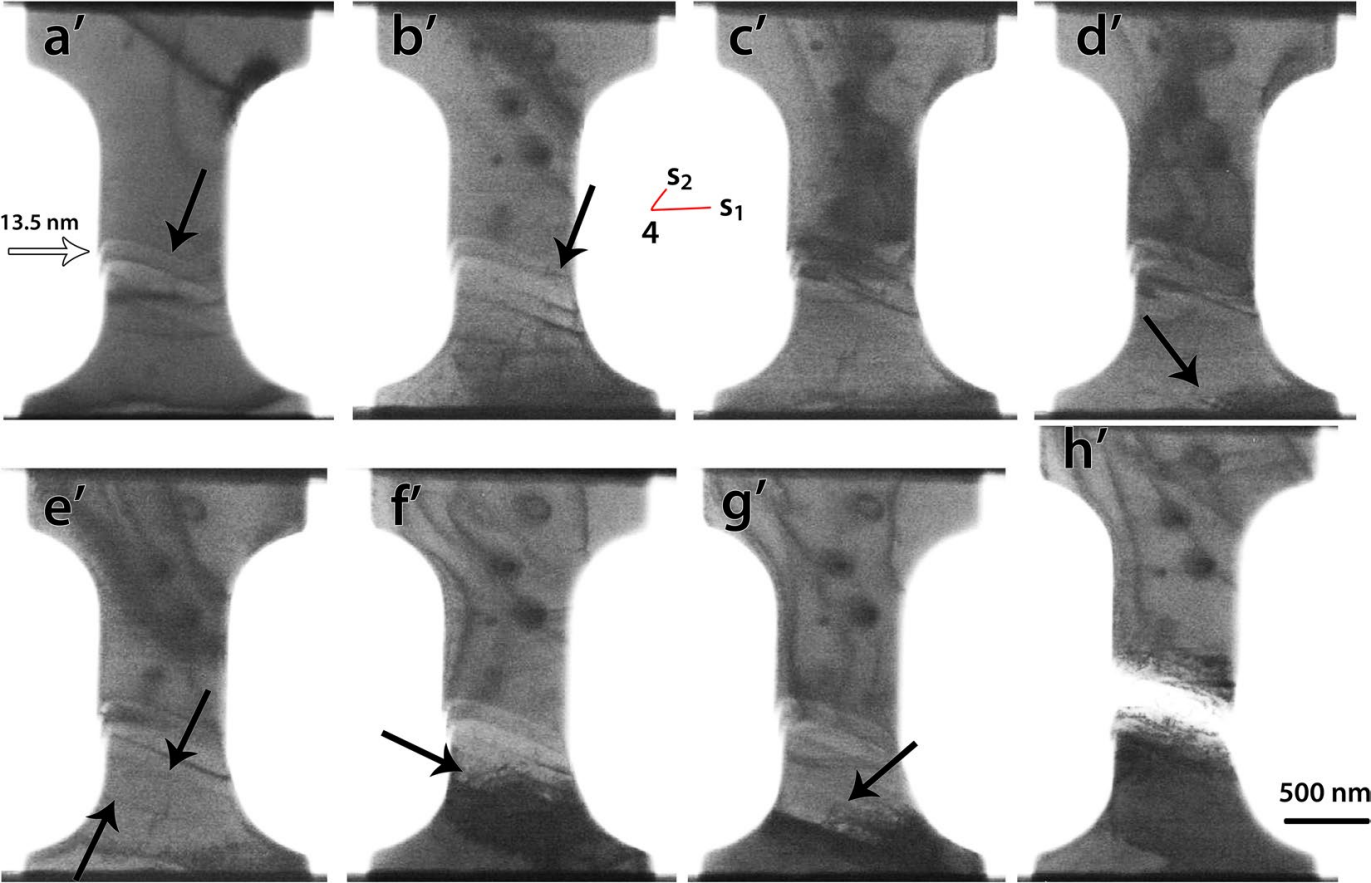
Snapshots from the deformation movie of the third cycle (**a’**), of the fourth cycle (**c’**–**e’**) and of the fifth cycle (**f’**–**h’**) in Fig. 3.

It is also worth noting that changes of the crystallographic orientation of the sample by 1–2° at the position of the STs shown in Fig. 4h and Fig. 6a’ was measured after cycle 3 (yellow plot in Fig. 3) using the automated crystallography orientation mapping in TEM (ACOM-TEM) technique (see SM)^61^. However, the effect of such a behaviour on the Schmid factor in the active slip system is very small (from 0.4686 to 0.4633). It can thus be concluded that the strain hardening in cycle 2 is mainly due to the shortening of the length of the source induced by the reduction of the slipped area with the accumulated plastic displacement. This is an important finding which provides a self-stabilization mechanism of SAS induced plasticity. Indeed, the absence of strain hardening is always favouring the occurrence of localization processes and earlier fracture (see e.g.^62^). Here it should also be noted that, because the theoretical calculations shown in Fig. 5 match very well with the experimental results without the need for including a contribution from frictional stress, the effect of Ga atoms (which might be present in the form of solid solution after annealing) on the plastic flow of the SAS cannot be considered as a dominant mechanism. However, the contribution of such feature in the high yield stress (compared to pure Ni) cannot be totally excluded since higher concentrations of Ga atoms might be present in the form of Cottrell atmosphere at the core of the initial dislocations.

In cycle 3, one strain burst occurred at 800 MPa and was accompanied by the formation of a new ST parallel to those formed by the operation of the SAS1 in cycle 2 (black arrow Fig. 6a’). This can be explained by the destruction of SAS1 upon unloading in cycle 2 and the activation of a new SAS2. Indeed, the imposed compressive force of the springs of the PTP on the sample and the slight buckling of the specimen after full unloading could destabilize SAS1. Figure 6b’ shows that, after unloading in cycle 3, the configuration of the red dislocation has changed in comparison with Fig. 4g. Indeed, only segment 4s_1_ connected to another segment 4 s_2_ can be observed while segments 34 and 3s disappeared. Therefore, it can be anticipated that point 3 was annihilated at the surface, leading to the formation of SAS2 with a new segment 4s_2_ rotating around pinning point 4. Based on the measurements of the total plastic displacement (14 nm) and the height of the ledge (13.5 nm) induced by the SAS2 in cycle 3 (white arrow in Fig. 6a’), it can be concluded that around 80 dislocations have been nucleated in the slip system $a/2\,[\bar{1}\,0\overline{\,1}]\,(1\,1\overline{\,1})$ (see SM). However, in contrast to cycle 2, no clear strain hardening can be observed in cycle 3 because of the setting of the experiment. Indeed, the maximum load imposed in this cycle (800 MPa) was very close to the load required to activate the SAS2. Thus, extra information on the plastic flow of this source cannot be extracted from this cycle.

In cycle 4 (purple plot in Fig. 3), a clear transition in the mechanical response occurred as evidenced by the drastic increase of the yield strength in this cycle (1030 MPa) compared to cycles 2 and 3. Furthermore, a more homogenous plastic flow and higher strain hardening capacity can be observed in cycle 4. In Fig. 6d’, dislocation pile-up gliding in the $(1\,1\overline{\,1})$ plane towards the bottom grip of the sample can be observed. The large black dots in Fig. 6 are contaminations on the surface formed during the ACOM-TEM alignment and measurements. New STs and other pile-ups of dislocations can also be seen after unloading (Fig. 6e’) and at the beginning of cycle 5 (Fig. 6f’). This confirms the shutdown of SAS2 in cycle 4 which can be explained by the cross-slip of the mobile segment 4 s_2_ out of the $(1\,1\overline{\,1})$ plane during unloading in cycle 3. The dislocation pile-ups shown in Fig. 6d’,f’ have been nucleated from the surface or from the dislocations left after annealing at the bottom grip of the sample (with a larger cross-section), indicated by the lower black arrow in Fig. 4a. This can be attributed to the absence of active dislocation sources within the gage section, leading to a significant increase of the yield stress after cycle 3. Surface nucleation might require higher stresses than observed in the present work^47^, but the contribution of such a feature cannot be totally excluded. The strain hardening observed in cycle 4 can be attributed to the plastic activity on the constrained $(1\,1\overline{\,1})$ slip planes at the bottom grip of the sample with the formation of dislocation pile-ups and the resulting back stress (i.e., kinematic hardening). Kiener *et al*. inferred the presence of dislocation pile-ups near the gauge section-grip interface in Cu samples after unloading by measuring the local crystal misorientations via electron backscatter diffraction (EBSD)^57^. These authors also reported that the additional strain hardening effect due to the tensile sample geometry vanishes when the aspect ratio (gauge length to width) is larger than 2. The sample used in the present study exhibits an aspect ratio of about 1, in agreement with the observation of dislocation pile-ups near the bottom grip of the PTP. In cycle 5 (green plot in Fig. 3), a decrease of the yield strength compared to cycle 4 was observed due to the lower stress needed to re-activate the glide of the pre-existing dislocations within the pile-ups as can be seen in Figs. 6f’,g’. Finally, the sample failed parallel to the $(1\,1\overline{\,1})$ slip plane (Fig. 6h’) at 1200 MPa. Cracking occurred at the ledge induced by the operation of the SAS2 in cycle 3 (see also SM).

In order to take advantage from the sample preparation method presented in this study, similar quantitative *in-situ* TEM tensile experiments on samples with pre-selected dislocation density and twin/grain boundaries (see Fig. S1 in SM) are under preparation. It is also worth noting that complete characterization of the nature of the defects and their 3D arrangement near the free surface before and during *in-situ* TEM deformation would open windows for more accurate comparison with DD and molecular dynamics (MD) simulations. In this context, recent advances of tilt-less 3D electron imaging and reconstruction of dislocations can be very promising. Such features will be addressed in future works.

## Conclusions

A new dedicated sample preparation method has been successfully used to investigate the mechanical response of a micron size pure Ni single crystal and to re-visit the mechanisms associated to the operation of single arm sources in small-scale single crystals. Almost defect-free samples allow observing the elementary discrete mechanisms controlling the nucleation and glide of dislocations near the free surface in a quantitative manner without the omnipresent artefacts of typical FIB prepared specimens. Furthermore, because dislocations induced by FIB could affect the strength and the plastic flow by acting as sources and obstacles for mobile dislocations, the present work brings new experimental evidences on the so-called staircase hardening behaviour of single crystals. Our results reveal that, in the absence of FIB damages, the initial relaxation of the few dislocations left after annealing leads to the formation of stable SASs. The lifetime of these sources was also increased (compared to similar sources in classic FIB prepared specimens) allowing detailed investigation of their intrinsic properties. An original strain hardening behaviour resulting from the operation of a SAS was observed. By combining the experimental observations with DD simulations, this was attributed to the shortening of the length of the source with the accumulated plastic displacement due to the reduction of the slipped area. A scenario accounting for the sample geometry evolution associated to SAS dynamics was proposed to explain the origin of the staircase behaviour observed in tensile test in the load control mode. It involves the contribution of a stress overshoot for the activation of the SAS. This discovery highlights the key importance, beyond standard crystal orientation and boundary definitions, of accurate 3D measures of the sample geometry evolution during micro-mechanical tests when applied to small samples.

## Methods

A high purity Ni foil (99.999%) (Goodfellow GmbH, Bad Nauheim, Germany) was annealed for 1 hour at 400 °C and then punched, ground and mechanically polished. The 3 mm discs were then electro-polished with a solution of perchloric acid and acetic acid, 1:4, in a Tenupol 3 instrument (Struers ApS, Ballerup, Denmark) at 0 °C, 18–19 V, and 100 mA. The electro-polished 3 mm discs were investigated by conventional TEM techniques in a Tecnai G2 TEM (FEI Company, Hillsboro, OR USA) operating at 200 kV in order to select proper regions in terms of thickness, electron transparency, crystallographic orientation, nature and density of defects, etc. A FEI Helios Nanolab 650 dual beam FIB/Scanning electron microscopy (SEM) was then used to cut out the tensile samples by the Ga^+^ ion beam at the appropriate locations. In order to minimize the FIB induced damage during the imaging by ion beam, the selected regions were minimally exposed to the FIB beam. Micron-sized dog-bone patterns were thus designed by FIB. One end of the dog-bones was cut like a spring shape (arrow in Fig. 1a) in order to accommodate possible thermal expansion and prevent bending of the samples during heat treatment. To ensure that the thickness along the tensile sample is uniform, the tensile axis of the samples was selected parallel to the edge of the electro-polishing hole or from regions where no clear thickness contrasts were observed. The amount of FIB damages and the span of these damages over the sample mainly depend on the FIB conditions, the required current/voltage and the cutting duration which vary also with the thickness of the electro-polished foil and the type of material. In general, the thicker the sample, the wider the affected zone because of the longer duration of the FIB exposure. In Fig. 1b, a TEM bright-field (BF) image of the dog-bone sample edge before heat treatment shows FIB induced defects.

After 30KV/80pA FIB cutting of the edges of the dog-bones by the designed pattern, the electro-polished sample was heat treated using a Gatan *in-situ* TEM heating holder for 5 min at 400 °C, 10 min 500 °C and 60 min at 700 °C in which the FIB damages have been removed. The main advantages of using the heating holder are the high-quality vacuum in the microscope and simultaneous observation of defect evolution during treatment^27^. During the *in-situ* TEM heating, it was observed that the FIB induced dislocations were either annihilated at the surfaces or coagulated with each other to form long dislocations and junctions, some of which again were annihilated at the surface. The rate of annihilation of dislocations in thin parts of the sample is faster than in thicker parts, which can be attributed to the effect of the image force^63^. In general, the final density of dislocations highly depends on the time and temperature of the heat treatment. Increasing both factors up to the values mentioned above, leads to large dislocation-free areas in the present Ni samples.

The commercial PI95 PicoIndenter instrument (Brucker.Inc) as well as a dedicated silicon Push-to-Pull (PTP) device were used to perform quantified *in-situ* TEM tensile testing, see Fig. 1c,d and ref.^22^. In Fig. 1d, compressing the half-circle dome by the flat punch indenter moves of the upper part downward while the lower part is stationary. The 4 identical device springs are distributed symmetrically at the corners so that the mounted sample across the gap of the PTP, red arrows in Fig. 1(d), is thus pulled in uniaxial tension. Following the *in-situ* TEM heat treatment, the dog-bone samples were cut and mounted on PTP devices using the Omniprobe (Oxford Instruments plc, Tubney Woods, UK) in the FIB instrument. Once the Omniprobe is attached by Pt deposition to one side of the dog-bone, as shown in Fig. 1c, the remaining edges of the dog-bones were cut from the sides, point A and B in Fig. 1c, by FIB. The samples were then mounted on PTP devices by Pt deposition on the sides, Fig. 1d, and then the Omniprobe was detached from the sample by FIB. Figure 1e shows the final configuration of a mounted sample on a PTP device. The significance is in the whole process of mounting, so great care was taken to avoid any exposure of the gages, the area C in Fig. 1c, by the ion beam. Conventional TEM revealed that FIB has not induced any extra defects during the mounting step of the samples, as seen in Fig. 1f. The chemical composition of one of the samples was checked by energy dispersive X-ray Spectroscopy (EDX) - Annular Dark Field scanning transmission electron microscope (ADF-STEM) in a FEI Osiris TEM operating at 200 kV. Figure 1h,i show the distribution of gallium and nickel in the sample. Quantitative analysis on different areas showed that the gallium amount in the sample is negligible (less than 1% which is below the EDX precision) so any gallium present after the FIB cutting of the shape of the sample^22^ has diffused during the annealing and formed a dilute solid solution. Ideally, the performed heat treatment to remove FIB induced defects should be performed of the entire setup after FIB mounting, but this is not possible due to a risk of diffusion of the platinum, used to clamp the sample onto the PTP device, into the MEMS device or into the sample at high temperatures.

The sample preparation procedure described here has several attractive advantages. Indeed, TEM investigation of the electro-polished sample before FIB cutting offers the possibility of an accurate crystallographic characterization and defect analyses which allows some freedom for prescribing the tensile sample condition in terms of loading direction, imaging conditions and crystallographic defects. Figure S1 (see SM) shows TEM-BF micrographs obtained on different samples including single crystal specimens with no dislocations or with a well-controlled population of initial dislocations as well as specimens with single interfaces such as grain boundaries or twin boundaries. In addition, the cleanliness of the samples allows improving the quality of the TEM observations of the intrinsic deformation/failure mechanisms as well as a quantitative correlation between these mechanisms and the load-displacement characteristics.

The *in-situ* TEM tensile experiments were carried out in load control mode with a loading rate of $2nN/s$ (initial strain rate of $2\times {10}^{-4}\frac{1}{{\rm{s}}}$) in a FEI Osiris TEM operating at 200 kV. The sample was mounted on a PTP device with a spring constant of 490 *N*/*s*. The length of the reduced section of the sample was 1.1 um, the width 800 nm and the thickness was around 300 nm (see SM for more details). According to the spring constant and the displacement data obtained from digital image cross-correlation of the deformation movies, the actual applied load on the sample, which is the subtraction of the spring load from the applied load, was calculated. The engineering stress (*σ*_*eng*_) was extracted by measuring the initial cross-section area of the gage length of the sample by FIB cross-sectioning after fracture. The uncertainty on the applied stress is mainly due to the precision of the measurement of the cross-sectional area of the tensile samples in the SEM images (~3 nm) as well as that of the force applied on the sample (~0.2 µN). Following the rules of propagation the standard error on the cross-sectional area is ~500 nm^2^ and on the engineering stress is ~3 MPa. Image cross-correlation of the deformation movies provided the gage length displacement data, allowing the calculation of the engineering strain (*ε*_*eng*_).

## Electronic supplementary material

Supplementary Materials
