# Supplementary material for: Dislocation driven nanosample plasticity: new insights from quantitative in-situ TEM tensile testing
Source: Sci Rep. 2018 Aug 13;8:12012. doi: 10.1038/s41598-018-30639-8 (PMC6089927; doi:10.1038/s41598-018-30639-8)
Supplement: Supplementary file 1 — Supplementary Materials [file 41598_2018_30639_MOESM1_ESM.pdf]

Dislocation driven nanosample plasticity: new insights from quantitative *in-situ* TEM tensile testing

Vahid Samaee[1]*, Riccardo Gatti[3], Benoit Devincre[3], Thomas Pardoen[2], Dominique Schryvers[1], Hosni Idrissi[2,1]

[1]Electron Microscop for Materials Science (EMAT), Department of Physics, University of Antwerp, Belgium
[2]Institute of Mechanics, Materials and Civil Engineering, Université catholique de Louvain, Belgium
[3]Laboratoire d'Etude des Microstructures, UMR104 CNRS-ONERA, 29 av. de la division Leclerc, Chatillon, France

*The corresponding author : Vahid.Samaeeaghmiyoni@uantwerpen.be

Supplementary material

Examples of samples with pre-selected microstructure

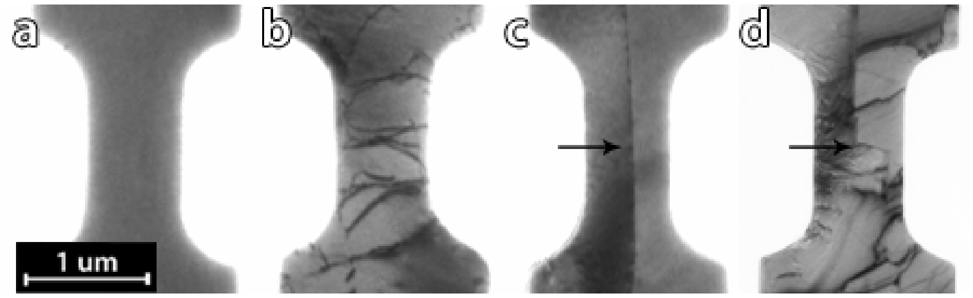

Fig. S1. TEM-BF micrographs of samples with different initial microstructure. (a) defect-free single crystal; (b) sample with 'high' initial density of dislocations; (c) sample with grain boundary; (d) sample with ∑3 111 twin boundary exhibiting a step indicated by black arrow. These samples experienced similar heat treatment procedure as the sample discussed in the paper.

Dimensions of the Sample

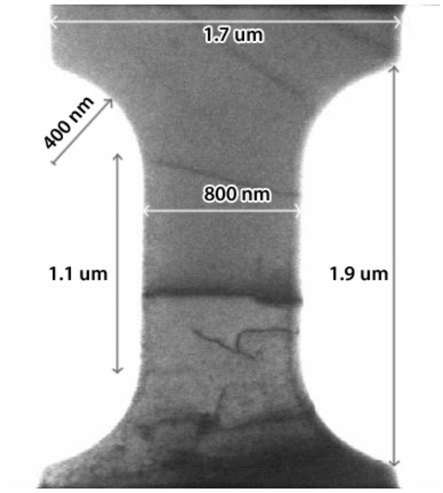

Fig. S2. Dimensions of the sample. The thickness was measured as 300 nm ± 16 nm by FIB cross-sectioning.

Comparison of slip traces in the first and second cycles of Fig. 3

Fig. S3 shows an overlapping of Fig. 4b and Fig. 4h. It shows that the STs induced by the blue dislocation (blue line in Fig. S3) are different from those made by the SAS1 (white dashed lines). Note that the blue dislocation has experienced cross-slip close to the surface.

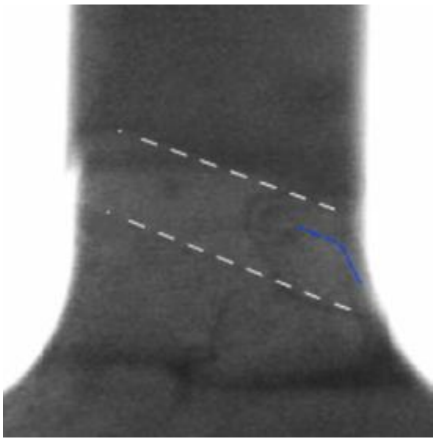

Fig. S3. Overlapping of Fig. 4b and Fig. 4h.

Magnified image of the SAS1

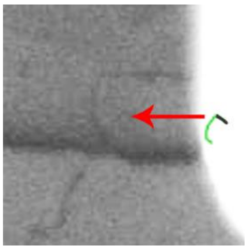

Fig. S4. Snapshot at point g of Fig. 3. The red arrow indicates the position of the active SAS1 in cycle 2.

Characterization of the slip system and the number of dislocations in cycles 2 and 3

The direction of the parallel STs made in cycle 2 (Fig. 4h) shows that the dislocations have glided on the $(1\,1\,\bar{1})$ plane. The corresponding Burgers vector can be determined based on the measurement of the height of the ledge and the total plastic displacement in cycle 2. The displacement induced by the activation of a specific slip system in a single crystal can be calculated using the following geometrical equation:

$$t = \alpha b \sin\lambda \cos\beta \qquad \text{(S1)}$$

where λ is the angle between the Burgers vector **b** and the normal to the foil surface; β is the angle between the Burgers vector's projection on the foil surface plane and the unknown displacement vector **t** while α is the number of dislocations.

The displacement along the tensile direction resulting from activation of the dislocation source was measured from the stress-strain curve in Fig. 3 as 31 nm while the final height of the step on the edge of the sample (i.e., normal to the tensile direction) measured from Fig. 4h equals 32 nm. These values confirm that the value of β for both cases (parallel and normal to the tensile direction) should be close to 45°. Among the slip systems involving the $(1\,1\,\bar{1})$ plane, only the one with Burgers vector $\frac{a}{2}[\bar{1}\,0\,\bar{1}]$ yields such value of β. Accordingly, λ equals 64°. By considering the Burgers vector $^{a}/_{2}\,[\bar{1}\,0\,\bar{1}]$, equation (1) reveals that around 200 dislocations have nucleated and reached the surface in cycle 2. In cycle 3, the displacement was around 14nm while the height of the ledge was

13.5 nm. Thus, around 80 dislocations have been nucleated in the active slip system $a/_2\,[\bar{1}\;0\;\bar{1}](1\;1\;\bar{1})$.

Reduction of the slipped plane area due to the operation of SAS1 in cycle 2

Fig. S5a shows the direction of $a/_2\,[\bar{1}\;0\;\bar{1}]$ Burgers vector as well as the direction $[2\bar{1}1]$ which is parallel to the intersection of the slip plane $(1\;1\;\bar{1})$ with the surface plane $(2\;\bar{1}\;\bar{5})$. The intersection of the slip plane $(1\;1\;\bar{1})$ with the surfaces of the sample delimits the slipped area. Let's consider the rectangle ABCD Fig. S5b as the area swept by one dislocation with Burgers vector $a/_2\,[\bar{1}\;0\;\bar{1}]$. The edges AD and BC correspond to the intersection of the slip plane with the top surface plane $(2\;\bar{1}\;\bar{5})$ while the edges AB and CD define the intersection of the slip plane with the sample surface on the sides. Fig. S5b shows that, after the glide of the dislocation, the slipped area decreases from ABCD to A'B'C'D. According to the slip geometry, the width of the slip area rectangle decreases by 0.5|b| while its length decreases by 0.86|b| (|b| = 0.24 nm). Therefore, the slip of around 200 dislocations at the end of the burst 4 of the cycle 2 would decrease the initial width (AB = 356 nm) and length (BC = 875 nm) of the rectangle by ~24 nm ($135 \times\; 0.5|b|$) and ~41 nm ($136 \times\; 0.86|b|$), respectively. Because of the shape of the sample, it is possible to assume that the dislocation source has the shortest length when its line is normal to either the AD or BC edge. Therefore, at the end of burst 4 the length of the dislocation source should decrease by ~24 nm. Furthermore, the decrease of the slip area leads to an increase of the RSS acting on the source. The corrected RSS at the end of the strain bursts in cycle 2 is shown in table S1.

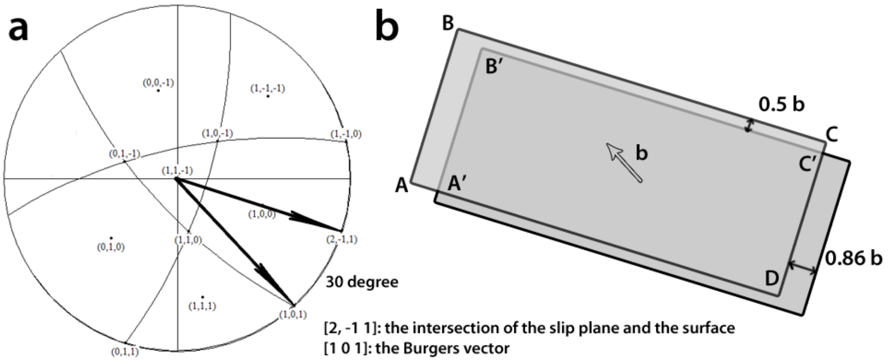

Fig. S5. (a) Stereographic projection showing the direction of intersection of the slip plane $(1\;1\;\bar{1})$ with the surface $(2\;\bar{1}\;\bar{5})$ and the Burgers vector, $a/_2\,[\bar{1}\;0\;\bar{1}]$, (b) schematic illustration showing the slipped area before (ABCD) and after the glide of one dislocation (A'B'C'D).

Table S1. Corrected RSS at the end of the bursts of cycle 2 when taking into account the decrease of the slip area

Burst	E-Stress (MPa)	RSS (MPa)	The corrected RSS at the end of the burst (MPa)
1	580	270.9	272.7
2	610	284.9	295.9
3	671	313.4	339.4
4	768	358.7	405.1

Evolution of τ_{area} with the source length shown in Fig. 5c.

According to the data shown in the previous section, it can be assumed that the length of the source *L* changes following the equation:

$$l = R - n(0.5\boldsymbol{b})$$

$$n\boldsymbol{b} = 2(R - l) \qquad \text{(S2)}$$

where *n* is the number of the nucleated dislocations, and *R* is the initial length of the SAS. On the other hand, the evolution of the shear stress due to the reduction of the slip area can be described using the following formula:

$$\tau_{area} = \tau_0 \times \frac{A_0}{A_{area}}$$

$$A_0 = 356\ (\text{nm}) \times 875\ (\text{nm})$$

$$A_{area} = (356 - 0.5\, nb) \times (875 - 0.86nb)$$

$$\tau_{area} = \tau_0 \times \frac{356 \times 875}{(356 - 0.5\, nb) \times (875 - 0.86nb)} \qquad \text{(S3)}$$

By combining Eq.(S2) and Eq.(S3), the following equation that describes the evolution of the shear stress as function of the length of the source can be obtained:

$$\tau_{area} = \tau_0 \times \frac{356 \times 875}{(356 - 0.5\,(2R - 2l)) \times (875 - 0.86(2R - 2l))} \qquad \text{(S4)}$$

The four grey curves in Fig. S6 (black dash curves in Fig. 5c) showing the evolution of τ_{area} with the source length were obtained using equation (S4) when $(\tau_0 = \tau_1, R = R_1)$, $(\tau_0 = \tau_2, R = R_2)$, $(\tau_0 = \tau_3, R = R_3)$ and $(\tau_0 = \tau_4, R = R_4)$. $\tau_{1,}\tau_{2,}\tau_3$ and τ_4 are the resolved shear stresses at the end of each burst that can be directly calculated using the Schmid factor from the engineering stress-strain curve reported in Fig. 3. These values have also been corrected by considering the slip area reduction effect at the end of the bursts in cycle 2 (see table S1). R_1, R_2, R_3 and R_4 are thus obtained from the red curve which corresponds to the equation (1) prediction for a screw dislocation.

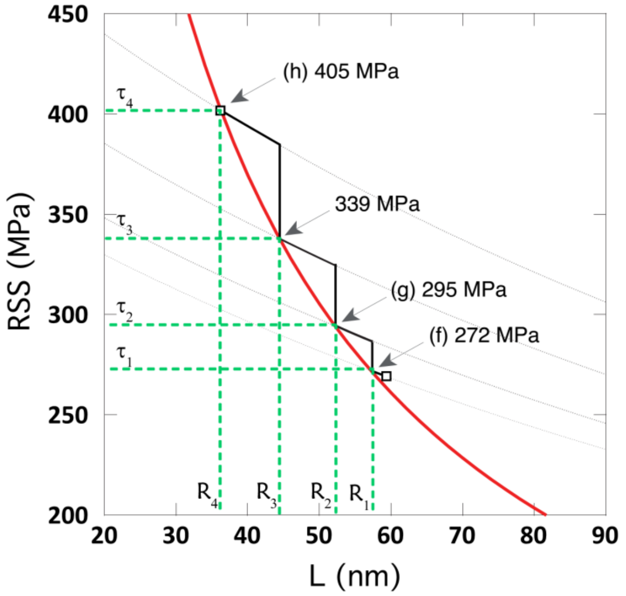

Fig. S6. Fig. 5(c) in the main text in which the different values of τ_0 and R are highlighted with green dash lines.

Local changes of orientation using ACOM-TEM

The ACOM-TEM technique was used in a Tecnai G2 TEM operating at 200 kV in order to generate orientation mapping. 30 μm C2 aperture, precession angle of 0.5°, probe size of 3-4 nm and step size of 50 nm have been used. It can be seen that, the crystallographic orientation of the sample changes at the position of the STs by 1°-2° due to the deformation constraints imposed by the PTP mounting setup. Dispersion of the measurement are due to the fact that, the measured misorientations fall within the angular resolution limits of the technique (around 1°).

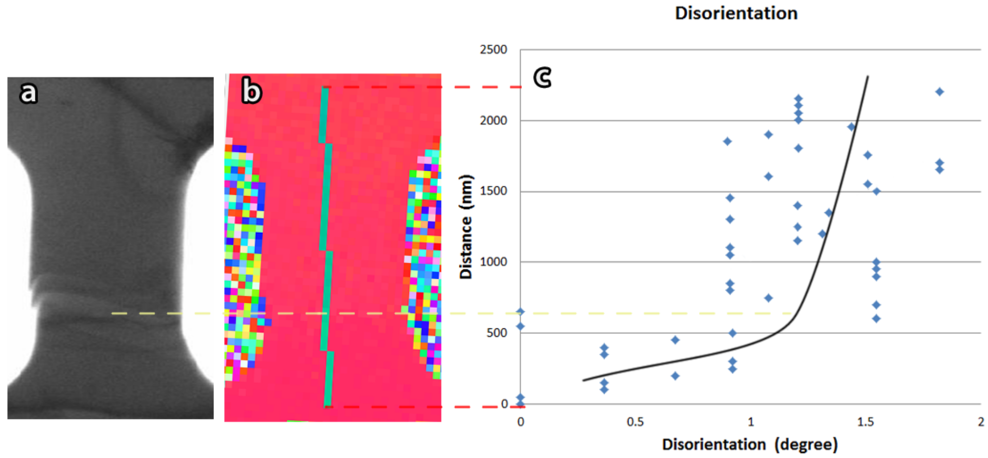

Fig. S7. (a) BF micrograph after cycle 3, (b) Orientation map after cycle 3 obtained by ACOM-TEM. (c) Corresponding orientation changes along the green vertical line in (b).

Fracture of the sample

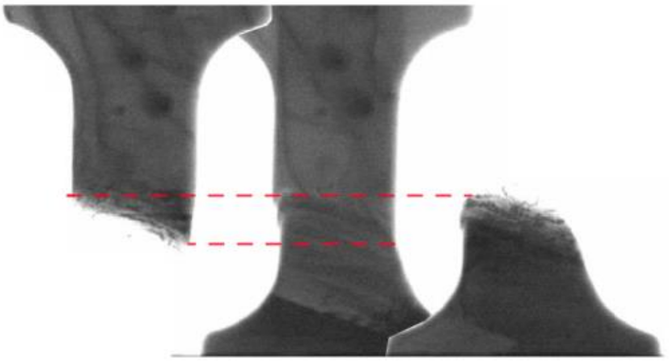

Fig. S8. Snapshots before and after the fracture of the sample. It can be seen that the fracture occurred along the slip plane from the upper step made by the SAS2, as indicated by the red dashed lines.
